# 多发性骨髓瘤6q缺失患者的临床特征研究

**DOI:** 10.3760/cma.j.issn.0253-2727.2021.08.005

**Published:** 2021-08

**Authors:** 蕾 陈, 春艳 孙, 博文 安, 建明 余, 菲 赵, 纯 张, 豫 胡

**Affiliations:** 1 华中科技大学同济医学院附属协和医院血液病研究所，武汉 430022 Institute of Hematology, Union Hospital, Tongji Medical College, Huazhong University of Science and Technology, Wuhan 430022, China; 2 信阳市中心医院血液内科，信阳 464000 Department of Hematology, Xinyang Central Hospital, Xinyang 464000, China

**Keywords:** 多发性骨髓瘤, 细胞遗传学, 6q缺失, 预后, Multiple myeloma, Cytogenetics, 6q deletion, Prognosis

## Abstract

**目的:**

研究多发性骨髓瘤6q缺失患者的临床特征、细胞遗传学特点，分析6q缺失对患者生存的影响。

**方法:**

回顾性分析华中科技大学同济医学院附属协和医院2014–2017年新诊断的382例多发性骨髓瘤患者，比较6q缺失患者与非6q缺失患者的临床特征及细胞遗传学特点。并采用Log-rank检验及Cox比例风险回归模型分析影响患者无进展生存（PFS）期和总生存（OS）期的预后因素。

**结果:**

6q缺失患者与非6q缺失患者比较，中位年龄较高（63岁对58岁，*P*＝0.039），t（4;14）发生率较高（30.4％对16.4％，*P*＝0.020），并伴有更高比例的复杂核型（22.2％对5.3％，*P*＝0.001）。Log-rank单因素生存分析显示，6q缺失与较短的PFS期相关。但在Cox多因素比例风险回归模型中，6q缺失不是独立的预后因素，其对生存期的影响受到年龄、t（4;14）等其他危险因素的影响。

**结论:**

多发性骨髓瘤中6q缺失多见于高龄患者，常伴随t（4;14）及复杂核型，但6q缺失不是独立预后因素。

## Abstract (en)

**Objective:**

To study the clinical and cytogenetic characteristics of patients with multiple myeloma harboring 6q deletion, with the aim to determine the impact of 6q deletion on survival.

**Methods:**

This study included the retrospective analysis of 382 newly diagnosed patients with multiple myeloma in our hospital from 2014 to 2017 and compared the clinical and cytogenetic characteristics between patients with and without 6q deletion. The log-rank test and the Cox proportional hazards regression model were used to analyze prognostic factors for progression-free survival（PFS）and overall survival（OS）.

**Results:**

Compared to those without 6q, the patients with 6q deletion were older（median age, 63 *vs* 58 years, *P*＝0.039）, had higher incidence of t（4; 14）（30.4％ *vs* 16.4％, *P*＝0.020）, and higher proportion of complex karyotypes（22.2％ *vs* 5.3％, *P*＝0.001）. Univariate survival analysis using the log-rank test revealed that 6q deletion was associated with shorter PFS. However, by the Cox multivariate proportional hazards regression model, 6q deletion was not an independent prognostic factor and its effect on survival was affected by age, t（4; 14）, and other risk factors.

**Conclusion:**

6q deletion was common in elderly patients with multiple myeloma and was often accompanied by t（4;14） and complex karyotypes. However, 6q deletion was not an independent prognostic factor for multiple myeloma.

多发性骨髓瘤（MM）是恶性浆细胞克隆性疾病，伴有多种复杂的染色体异常是其主要特征之一。细胞遗传学改变与MM的发病机制和疾病进展密切相关，已证实17p缺失、t（4;14）、t（14;16）、t（14;20）等是高危细胞遗传学异常，预示着较短的生存期[1]–[7]。除了这几种已明确的高危细胞遗传学改变，MM的染色体异常几乎涉及每一条染色体，其数量改变是最常见的异常。30％～40％的MM患者有多倍体异常，通常涉及多条奇数染色体的扩增。缺失则多见于13、14、16和22号染色体单体和一些染色体片段，如1p、6q、8p、12p、14q、16p和20p等[8]–[10]。6q缺失出现在10％～30％的MM患者中，是与亚二倍体有密切相关性的后续事件，通常伴随复杂核型[11]。MM中6q缺失患者的临床特征鲜有文献报道。为了解6q缺失MM患者的临床特征和生存情况，我们总结了我院2014至2017年间初治MM患者的临床资料，分析了6q缺失患者的临床特点。

## 病例与方法

1. 病例：本研究回顾性分析了华中科技大学同济医学院附属协和医院2014至2017年初次治疗前接受间期FISH（iFISH）检查的382例新诊断MM患者，诊断根据中国MM诊治指南（2013年修订版）[12]。MM患者中46例有6q23缺失，其中26例男性，20例女性。

2. 治疗：382例新诊断MM患者均使用以硼替佐米和（或）来那度胺为主的治疗。化疗方案包括VD方案（硼替佐米+地塞米松）、RD方案（来那度胺+地塞米松）、PAD方案（硼替佐米+阿霉素+地塞米松）、VTD方案（硼替佐米+沙利度胺+地塞米松）、VCD方案（硼替佐米+环磷酰胺+地塞米松）、RVD方案（来那度胺+硼替佐米+地塞米松）。48例（12.6％）患者接受了自体造血干细胞移植。

3. iFISH分析：在进行iFISH检测前，先将骨髓样本进行CD138磁珠（德国Miltenyi公司产品）分选，获得的浆细胞比例达85％以上。iFISH的操作按照说明书步骤进行。我们使用以下探针检测染色体异常：RB1（13q14）、P53（17p13）、CKS1B（1q21）/CDKN2C （1p32）、IGH （14q32）、MYB（6q23）、IGH/CCND1［t（11;14）］、IGH/FGFR3［t（4;14）］（美国Abbott公司产品），每个探针至少评估了200个细胞。

4. 随访：随访截止日期为2020年6月30日，中位随访时间33（12～57）个月，通过查阅患者住院病历、门诊随访记录和电话随访记录随访。无进展生存（PFS）期定义为从确诊至疾病进展、死亡或随访截止的时间。总生存（OS）期定义为从确诊至患者死亡或随访截止的时间。

5. 统计学处理：本研究所有的统计学分析均使用SPSS软件（IBM，version 26.0）完成。计量资料用*M*（*P*_25_，*P*_75_）表示。组间分类变量的比较采用卡方检验或Fisher精确概率法。连续性变量的比较使用Mann-Whitney *U*检验。与PFS和OS相关的单因素危险分析采用Log-rank检验，多因素分析采用Cox比例风险回归模型。*P*<0.05为差异有统计学意义。

## 结果

1. 临床特征：382例新诊断的MM患者中，46例（12％）有6q23缺失，其中男26例（56.5％），女20例（43.5％），中位年龄为63（57，70）岁，患者的临床特征见表1。6q缺失患者的中位年龄为63（57，70）岁，无6q缺失患者的中位年龄为58（52，65）岁，两组的差异有统计学意义（*P*＝0.039）。在ISS分期及R-ISS分期系统中，6q缺失患者与无6q缺失患者各分期比例的差异无统计学意义。此外，6q缺失患者发生贫血、PLT减低、LDH升高、高钙血症、血肌酐升高的比例与无6q缺失患者相比差异无统计学意义（表1）。

**表1:** 有、无6q缺失的多发性骨髓瘤患者临床特征比较［例（％）］

临床特征	有6q缺失（46例）	无6q缺失（336例）	*χ*^2^值	*P*值
性别			0.494	0.482
男	26（56.5）	208（61.9）		
女	20（43.5）	128（38.1）		
ISS分期			4.430	0.109
Ⅰ	10（21.7）	103（30.7）		
Ⅱ	15（32.6）	66（19.6）		
Ⅲ	21（45.7）	167（49.7）		
R-ISS分期			2.027	0.363
Ⅰ	8（17.4）	91（27.1）		
Ⅱ	24（52.2）	159（47.3）		
Ⅲ	14（30.4）	86（25.6）		
HGB < 85 g/L	22（47.8）	178（53.0）	0.430	0.512
PLT < 100×10^9^/L	14（30.4）	63（18.8）	3.433	0.064
LDH ≥ 245 U/L	10（21.7）	39（11.6）	3.715	0.054
血清钙 ≥ 2.65 mmol/L	8（17.4）	37（11.0）	1.584	0.208
血肌酐 ≥ 20 mg/L	6（13.0）	73（21.7）	1.860	0.173

2. 细胞遗传学特点：在46例6q缺失患者中，44例患者伴随其他染色体异常，2例患者仅检出6q缺失。染色体核型分析发现6q缺失患者伴复杂核型（3种以上染色体异常）的比例较无6q缺失患者高（22.2％对5.3％，*P*＝0.001）。iFISH检测提示的其他伴随细胞遗传学异常见表2。6q缺失患者与无6q缺失患者13q缺失（52.2％对40.8％，*P*＝0.142）、17p13缺失（19.6％对12.8％，*P*＝0.209）、1p32缺失（17.4％对11.9％，*P*＝0.292）、1q21扩增（56.5％对50.0％，*P*＝0.407）及IGH易位（50.0％对50.6％，*P*＝0.940）的发生率相似。但6q缺失患者伴t（4;14）的比例较无6q缺失患者更高（30.4％对16.4％，*P*＝0.020）。此外，6q缺失患者t（11;14）的发生率低于无6q缺失患者，但两者的差异无统计学意义（8.7％对19.9％，*P*＝0.071）。

**表2:** 6q缺失多发性骨髓瘤患者的细胞遗传学异常［例（％）］

细胞遗传学异常	检测阈值	6q缺失（46例）	无6q缺失（336例）	*χ*^2^值	*P*值
13q缺失	10％	24（52.2）	137（40.8）	2.157	0.142
17p13缺失	10％	9（19.6）	43（12.8）	1.576	0.209
1p32缺失	10％	8（17.4）	40（11.9）	1.109	0.292
1q21扩增	5％	26（56.5）	168（50.0）	0.689	0.407
IGH易位	5％	23（50.0）	170（50.6）	0.006	0.940
t（11;14）	5％	4（8.7）	67（19.9）	3.381	0.071
t（4;14）	5％	14（30.4）	55（16.4）	5.409	0.020
复杂核型（G显带分析）	无	10（22.2）	18（5.3）	14.342	0.001

3. 生存分析：截至随访结束，75例（19.6％）患者死亡，其中6q缺失患者中有16例死亡，无6q缺失患者有59例死亡。6q缺失患者的死亡率高于无6q缺失患者（34.8％对17.6％，*P*＝0.006）。Log-rank单因素危险分析显示，6q缺失患者的中位PFS期较无6q缺失患者缩短（28个月对未达到，*P*＝0.018），但中位OS期与无6q缺失患者相比差异无统计学意义（47个月对48个月，*P*＝0.120）。

多因素分析显示，6q缺失不是PFS的独立危险因素（*P*＝0.220），而年龄（*P*<0.001）、17p缺失（*P*<0.001）、t（4;14）（*P*＝0.030）是PFS的独立危险因素（表3）。6q缺失也不是OS的独立危险因素（*P*＝0.766）。年龄（*P*<0.001）、17p缺失（*P*<0.001）、t（4;14）（*P*＝0.001）是OS的独立危险因素（表3）。

**表3:** 影响多发性骨髓瘤患者无进展生存和总生存的多因素分析

因素	无进展生存		总生存	
	*HR*（95％*CI*）	*P*值	*HR*（95％*CI*）	*P*值
年龄	1.065（1.039～1.093）	<0.001	1.116（1.084～1.150）	<0.001
17p缺失	5.918（3.176～11.027）	<0.001	8.163（4.313～15.448）	<0.001
1q21扩增	1.724（0.955～3.110）	0.071	1.365（0.750～2.484）	0.308
6q缺失	1.475（0.792～2.747）	0.220	1.105（0.571～2.141）	0.766
t（4;14）	1.951（1.067～3.567）	0.030	2.889（1.547～5.395）	0.001
t（11;14）	1.430（0.768～2.666）	0.260	1.070（0.555～2.064）	0.840

## 讨论

MM是浆细胞的恶性增殖性疾病，常伴随复杂的染色体异常。6q缺失是MM中一种常见的染色体缺失异常，发生率为10％～30％，在其他淋巴系统恶性增殖性疾病中也较常见。在滤泡性淋巴瘤中，6q缺失发生率为25％～30％，与不良预后相关[13]。华氏巨球蛋白血症中6q缺失发生率为40％左右，但与预后无明显相关性[14]。CLL中6q缺失的发生率大于20％，与FOXO3低表达相关，是预后不良因素[15]–[16]。虽然6q缺失的预后意义尚不统一，但6q缺失在多种恶性疾病中普遍出现，提示6q缺失与肿瘤发生发展密切相关。

本研究382例MM初治患者中332例（86.9％）经iFISH检测发现了不同程度的染色体异常，其中46例（12％）有6q缺失。6q缺失患者的年龄较无6q缺失患者大，性别、临床特征、靶器官损伤等的差异均无统计学意义。但6q缺失患者合并复杂核型的发生率较高，且伴随较高的t（4;14）发生率和似乎更低的t（11;14）发生率。

截至本研究随访结束，6q缺失患者有较高的死亡率。Log-rank单因素分析显示，6q缺失与较短的PFS期相关，与OS无明显相关性。但在多因素分析中，6q缺失不是独立的预后危险因素，提示6q缺失的预后意义受到其他多重因素的影响，如年龄较大、合并t（4;14）及复杂核型等。

6q缺失的分子机制尚不明确，多项研究显示，6q缺失存在多种模式，涉及不同的染色体区域和基因。Thelander等[17]在49例包含套细胞淋巴瘤、弥漫大B细胞淋巴瘤、滤泡性淋巴瘤转化的弥漫大B细胞淋巴瘤及儿童急性淋巴细胞白血病患者中发现26例6q缺失患者，其中85％为6q21中3 Mb的片段，涉及FOXO3A、PRDM1和HACE1基因，还有两例弥漫大B细胞淋巴瘤患者为6q23-24纯合缺失，涉及TNFAIP3基因。Gachet等[18]发现，在T细胞急性淋巴细胞白血病患者中，6q14缺失导致两个连续基因SYNCRIP和SNHG5缺失，从而通过核糖体-线粒体轴的失控增强肿瘤细胞代谢能力，促进T细胞急性淋巴细胞白血病的发展。而在T淋巴母细胞淋巴瘤中，6q15-6q16.3的缺失片段定位了抑癌基因Epha7，其下调促进了T淋巴母细胞淋巴瘤的发展[19]。

6q缺失是包含多个区域和基因的较复杂的染色体改变，MM中6q缺失也可能涉及不同的染色体区域，导致相关基因异常而促进肿瘤发生发展，但目前无相关文献报道。本研究初步探讨了6q23缺失的MM患者的临床特征，6q的其他区域缺失及其分子机制仍需要进一步探索。
